# The Evolution of the Isolation Hospital
*Prevous articles appeared on Dec. 6 and Nov. 22, 1913.


**Published:** 1914-01-10

**Authors:** H. Franklin Parsons

**Affiliations:** late Assistant M.O. L.G.B.


### THE EVOLUTION OF THE ISOLATION HOSPITAL.*

By the late H. FRANKLIN PARSONS, M.D., D.P.H., late Assistant M.O. L.G.B.

Both in London and elsewhere there have been considerable changes as regards the kind of cases admitted into isolation hospitals since they were first instituted. As already mentioned, isolation hospitals were at first provided especially for the graver and more formidable epidemic diseases, such as smallpox, cholera, typhus, and relapsing fever —diseases which are now extinct or only rarely threaten at long intervals. As they declined the hospitals were used for scarlet fever, formerly a much more formidable disease than it usually is at the present day. Diphtheria and enteric fever cases were formerly treated in general hospitals, but as their infectious nature became recognised the practice of receiving them into general hospitals gradually ceased, and they came to be regarded as proper subjects for the isolation hospital. Smallpox required isolation, but as it was found that there was risk of its spreading to patients with other diseases if treated in the same hospital with them separate smallpox hospitals have been provided in the larger districts. Scarlet fever, diphtheria and enteric fever have thus been the diseases most frequently treated in isolation hospitals, and of these scarlet fever has been the chief; in most hospitals it constitutes the majority of the cases; some local authorities have reserved their hospitals exclusively for it to the exclusion of other graver diseases, and some on the occasion of an epidemic have endeavoured to get every case into hospital, with the result of causing crowding detrimental to the patients. These practices have produced a reaction, and it has been urged by objectors that the practice of hospital isolation has not appreciably reduced the prevalence of scarlet fever; that as it is now a mild disease with a low mortality, the public in spending large sums in its isolation and treatment are not getting value for their money; and that the aggregation of cases in hospitals is a source of dangers such as cross-infection, septic complications, and prolonged infectiousness, leading to the occurrence of "return cases" on the patient's discharge. The chief reasons why hospital isolation has not effected a greater diminution in the number of cases of scarlet fever appear to be that in the present mild phase of the disease it is often contracted from slight cases which have escaped recognition; and that removal to hospital often does not take place until after an interval of time from the commencement of the case sufficient to allow other susceptible persons in the household to contract the disease.

In quite recent years cases of measles and whooping-cough, and also of puerperal fever, have been admitted into some isolation hospitals in large towns. Another use to which isolation hospitals, especially smallpox hospitals, which are often empty for long periods, have recently been put has been for the reception of cases of pulmonary tuberculosis. This may become frequent as a means of affording "sanatorium benefit" under the National Insurance Act.

The principles of construction of isolation hospitals have not undergone any very great change since Sir R. Thorne's Report thirty years ago. As already said, a larger provision of accommodation for staff and laundry and other appliances is now found necessary than was formerly deemed sufficient; and the advantage of combination of districts for the provision of well-equipped permanent hospitals of reasonably large size over the provision of small separate hospitals for individual districts has become more generally recognised. It is also recognised that hospitals require, in addition to larger wards for the treatment of multiple cases of one disease, small wards for the isolation of mixed or doubtful cases. One form which such wards have taken is that of the cubicle or glass cell, in which the patient while more or less completely separated aërially from his neighbours can nevertheless see them, and is under easy supervision by the nurse. Blocks of wards of this kind have been erected at several of the Metropolitan Asylums Board's hospitals, and at the Walthamstow, East Ham, Croydon Borough, Croydon Rural, and other hospitals. In some of these the compartments are completely separated aërially from one another and entered from the open-air under a verandah. In others they are entered from a central corridor, but otherwise completely separated; and in others, again, they are only partially separated by glazed partitions not reaching the ceiling. In either case careful nursing on aseptic lines is necessary in order to prevent risk of cross-infection, and it is held by some hospital physicians that such nursing is sufficient for the purpose by itself without aërial separation. This involves the need for a high standard of efficiency on the part of fever nurses. There is reason to think that in infectious diseases the primary infection is commonly accompanied by secondary infections—as scarlet fever by diphtheria bacilli or septic streptococci. It follows that a patient suffering from an infectious disease should be not only isolated from susceptible persons in health but also protected from cross-infection. This principle is called "bed-isolation."

Other modern methods which have been introduced are eucalyptus inunction and open-air treatment. The former, conjoined with swabbing of the throat with carbolic oil, is recommended by Dr. Milne in scarlet fever with a view to avert complications and cut short infectiveness; hence, it is claimed, rendering hospital isolation unnecessary. Opinions are divided as to the value of this method, but there is difficulty elsewhere than in an institution in applying it from the very commencement of the illness as Dr. Milne requires.

The open-air method is recommended by Dr. Boobbyer and others in cases of scarlet fever and diphtheria, especially for those with septic complications.

* Prevous articles appeared on Dec. 6 and Nov. 22, 1913.

